# Access to healthcare for people living with HIV: an analysis of judgments of the European Court of Human Rights from an ethical perspective

**DOI:** 10.3389/fpubh.2023.1193236

**Published:** 2023-06-12

**Authors:** Tobias Skuban-Eiseler, Marcin Orzechowski, Florian Steger

**Affiliations:** ^1^Institute of the History, Philosophy and Ethics of Medicine, Faculty of Medicine, Ulm University, Ulm, Germany; ^2^kbo-Isar-Amper-Klinikum Region München, Munich, Germany

**Keywords:** access to healthcare, people living with HIV, medical ethics, international law, human rights, stigmatization

## Abstract

**Introduction:**

Although HIV has been part of our reality for over 30 years, people living with HIV (PLHIV) still experience restrictions regarding their access to healthcare. This poses a significant ethical problem, especially as it endangers achieving the goal of ending the HIV epidemic worldwide. The aim of this paper is to analyze the rulings of the European Court of Human Rights (ECtHR) regarding cases where PLHIV experienced restrictions on their access to healthcare.

**Methods:**

We conducted an analysis of the ECtHR database and were able to identify *N* = 28 cases dealing with restricted access to healthcare for PLHIV. A descriptive and thematic analysis was conducted to identify ways in which access to healthcare for PLHIV was restricted.

**Results:**

We were able to identify a total of four main categories, with denial of adequate therapeutic support as the main category with *N* = 22 cases (78.57%). Most of the judgments examined were filed against Russia (*N* = 12, 42.86%) and Ukraine (*N* = 9, 32.14%). A large proportion of PLHIV in the cases studied (*N* = 57, 85.07%) were detainees.

**Discussion:**

The analysis shows a clear condemnation of limited access to healthcare for PLHIV by the ECtHR. Ethical implications of the analyzed cases are discussed in detail.

## 1. Introduction

When the disease that would become known as AIDS was first discovered in 1981 (1), there was no way of knowing the scope of its impact. In 2021, 38.4 million people worldwide were infected with HIV and 1.5 million people were newly diagnosed (2). The successes in combating the virus were and are enormous: the new retrovirus could be isolated as early as 1983, commercial HIV tests were available in 1984 and the first effective drug, azidothymidine, had been available since 1987 (1). Today, highly effective antiretroviral therapies are utilized to keep the viral load below the detection limit for a long time (3). Yet, only 85% of infected people know their HIV status, 9.7 million infected people do not have access to antiretroviral therapy and only 68% of infected people are virally suppressed (2). The HIV epidemic touches numerous aspects that require a medico-ethical discourse (4, 5). Considering the number of affected people, limited access to healthcare for many people living with HIV (PLHIV) remains a very relevant challenge for society and healthcare systems. Limitation of access to healthcare for PLHIV manifests itself in various ways, e.g., through negative interactions with medical staff, long waiting times, inability to pay for medical treatment, fear of being identified as HIV-positive, long distances to treatment centers, poor quality of treatment, etc. (6–8). Reasons for these phenomena are often contingent on the social and financial situation of PLHIV. Factors that play an important role include socio-demographic aspects (age of the infected person, ignorance of an HIV-positive person in the family, belonging to the female sex, being black, less than primary education, etc.), clinical aspects (low adherence to medication, experience of side effects of therapy, etc.), socio-economic factors (costs of therapy, food insufficiency, financial problems, etc.), health behavior (unprotected sex, non-disclosure of HIV-status, alcohol and drug abuse, etc.) and factors regarding the health-system (stock-outs of medication, inadequate communication with health staff, etc.) (9). Moreover, internalized and anticipated stigma has emerged as a significant factor hindering access to healthcare for PLHIV (9–11). Therefore, the reduction of stigma and discrimination has been formulated as one of the UNAIDS targets for 2025 (12). Stigma and discrimination also take place in the context of the health systems (11), for example through delays in specific interventions or unnecessary referrals to specialists (13). In this context, they are regarded as a major barrier to health-related quality of life for PLHIV (14) and are a significant factor preventing the achievement of global goals to end the HIV epidemic (15). Limited or even delayed access to healthcare leads to increased mortality and morbidity of PLHIV (16), raised healthcare costs (17) and a surge of risk of infecting other people (18). It is therefore of high general interest that PLHIV experience the same access to healthcare as non-infected individuals. Since PLHIV are not necessarily recognizable as suffering from an infection, and since access to healthcare for this group also includes the unrestricted possibility of appropriate infection testing as well as access to antiretroviral drugs, this work will focus on PLHIV and not explicitly on people suffering from AIDS.

Legal frameworks play an important role in guaranteeing equal access to healthcare for PLHIV and in their protection against discrimination and stigmatization in the healthcare system. In this context, jurisdiction and ethical reflection are closely linked and interrelated, as laws should at best reflect the moral considerations of a society.

In this paper, we address the ethical and legal discourses on restriction of access to healthcare for PLHIV. For this purpose, we conducted a systematic analysis of judgments of the European Court of Human Rights (ECtHR) dealing with restricted access to healthcare for PLHIV. The deliberations of the ECtHR included in judgments provide not only a legal perspective on the issue from the point of view of protection of Human Rights but also reflections on ethical perspectives. Therefore, they are especially valuable for analysis of issues related to discrimination and abuse of individuals from minority groups. As has been shown previously, systematic categorization and assessment of ECtHR’s judgments allows a better understanding and illustration of medico-ethical and legal discourses on discrimination of vulnerable groups in healthcare (19–23). Moreover, since 46 Member States of the Council of Europe can request the ECtHR to review a case, the analysis of the Court’s proceedings provides valuable information on offenses against Human Rights in the Member States of this organization.

In our research, we pursued the following questions: (1) how many ECtHR judgments deal with access to healthcare for PLHIV? (2) How can these judgments be grouped thematically in terms of different ways in which access to healthcare is restricted? (3) Can a particular vulnerable group be identified? and (4) What is the ECtHR’s position on these cases, particularly regarding the ethical aspects associated with them?

## 2. Materials and methods

**Figure 1:**
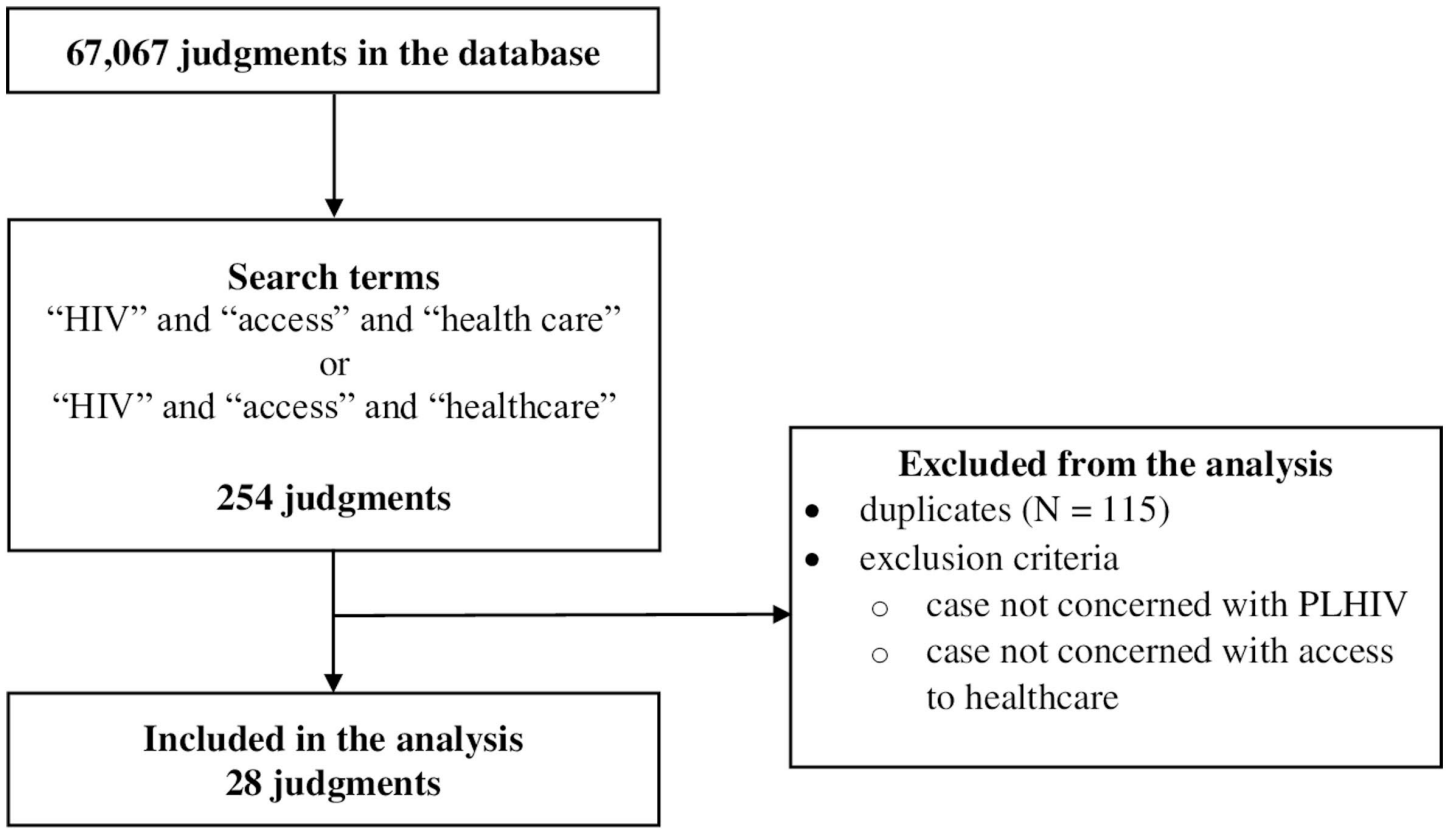
Flowchart of the search.

To identify judgments relevant for our analysis, we searched HUDOC, an online database of the ECtHR’s case law. We used standard settings of the HUDOC’s search engine https://hudoc.echr.coe.int/eng#{%22documentcollectionid2%22:[%22GRANDCHAMBER%22,%22CHAMBER%22]}. The search was performed on January 29, 2023, using the following search terms: “HIV,” “access” and “health care” or “HIV,” “access” and “healthcare.” The search yielded *N* = 254 results. After elimination of *N* = 115 duplicates, the remaining 139 cases were evaluated. Excluded were *N* = 111 cases that were either not concerned with PLHIV or were concerned with PLHIV but not with their access to healthcare. *N* = 28 relevant cases were included in the final analyzes (Figure 1).

### 2.1. Descriptive statistics

As a first step of the analysis, we performed descriptive statistics on the countries against which the applications were lodged, demographics of the individuals involved in the applications, and the Articles of the European Convention of Human Rights involved in the judgments. In the majority of the cases under deliberation, the ECtHR ruled on several articles in one case or only on particular paragraphs or aspects of a single article. For example, several analyzed judgments involve violation of a one sub-paragraph of an article or the court stated in its decision that the substantive aspect of an article was violated, while there was no breach of a procedural aspect. For our analysis, counted were violations of articles if at least one of their sub-paragraph or aspect were breached, even if the court held no violation of other sub-paragraphs or aspects of the involved articles.

### 2.2. Thematic analysis

In the following step, we performed a thematic analysis of all *N* = 28 relevant judgments. Thematic analysis is a qualitative approach used for identification and analysis of common or recurring themes or patterns in text materials (24, 25). Relevant parts of the analyzed judgments were manually coded, extracted, and compared. Based on this procedure, we inductively formulated 4 main thematic categories and 8 sub-categories. These categories were critically discussed in the multi-professional team including a psychiatrist (T.S.), a physician and expert in the history, philosophy and ethics of medicine (F.S.), and a medical ethicist and political scientist (M.O.). These categories represent important thematic patterns involved in judgments of the ECtHR regarding the research questions. The categories are not mutually exclusive; therefore, several analyzed cases were grouped into more than one category. Each of the categories is illustrated by a representative case report and provides an insight into the situations of limited access to healthcare for PLHIV.

## 3. Results

### 3.1. Countries against which applications were filed

The analyzed judgments involved applications against *N* = 6 countries. *N* = 12 applications were filed against Russia (42.86%), *N* = 9 against Ukraine (32.14%), *N* = 2 against each Romania, United kingdom and Greece (7.14% each), *N* = 1 against Latvia (3.57%).

### 3.2. Demographic characteristics of applicants

The examined *N* = 28 judgments deal with a total of *N* = 67 PLHIV. In *N* = 2 cases, the applicants involved in the case are not PLHIV. In these instances, we have only considered the PLHIV with whom these cases are concerned. In the analyzed judgments there is a clear dominance of the number of male PLHIV (m:*f* = 61:6). *N* = 57 applicants (85.07%) were people in detention. The age range of PLHIV in the analyzed judgments ranged from 20 to 63 (mean 28.56 years), although for 20 applicants it was not possible to determine their age. *N* = 18 PLHIV also suffered from other infections: *N* = 13 had viral hepatitis and *N* = 11 had tuberculosis (Table 1).

**Table 1:** Demographics of the PLHIV in the analyzed cases.

number of PLHIV in analyzed cases	67
m:f	61:6
detainees	57
age spectrum	20–63 (mean 28.56)
additional infections	18 (13 viral hepatitis, 11 tuberculosis)

### 3.3. Violation of articles of the European Convention on Human Rights

Only in *N* = 2 cases (7.14%), the ECtHR found no violation of any Article of the European Convention on Human Rights (appl. no. 26565/05, N. v. the United Kingdom and appl. no. 76317/11, Bubnov v. Russia). In the remaining *N* = 26 cases (92.86) the ECtHR found at least one violation of a total of eight Articles of the European Convention on Human Rights. The article that has been found to be breached in most cases is Article 3 (*N* = 20, 71.43%), followed by Article 13 (*N* = 8, 28.57%) and Article 5 (*N* = 6, 21.43%) (Table 2).

**Table 2:** Articles of the European Convention on Human Rights that were violated in the analyzed judgments.

Article of the European Convention on Human Rights	Name of Article	Number of cases in which Article has been found to be violated
Article 2	Right to life	*N* = 4 (14.29%)
Article 3	Prohibition of torture	*N* = 20 (71.43%)
Article 5	Right to liberty and security	N = 6 (21.43%)
Article 6	Right to a fair trial	*N* = 1 (3.57%)
Article 7	No punishment without law	*N* = 1 (3.57%)
Article 8	Right to respect for private and family life	*N* = 5 (21.43%)
Article 13	Right to an effective remedy	*N* = 8 (28.57%)
Article 14	Prohibition of discrimination	*N* = 3 (10.71%)

### 3.4. Categories of restricted access to healthcare

We were able to identify several ways in which PLHIV are denied access to healthcare. We formulated a total of four main thematic categories (each with a different number of subcategories) describing the limitations in access to healthcare experienced by PLHIV. These categories are: (I) refusal of diagnostic procedures (*N* = 7, 25%), (II) failure of providing information about HIV testing (*N* = 3, 10.71%), (III) refusal of adequate therapeutic support (*N* = 22, 78.57%) and (IV) administrative failures (*N* = 10, 35.71%). Some of the analyzed cases could be assigned to several categories (Table 3).

**Table 3:** Categories and subcategories of limitations of access to healthcare in the analyzed judgments.

Category	Subcategory	Number of cases	Example described in the text
Refusal of diagnostic procedures	Denial of HIV-test or diagnostic consequences of positive HIV-test	*N* = 2 (28.57%)	Logvinenko v. Ukraine (appl. no. 13448/07)
	Denial of necessary examinations in context of HIV-infection	*N* = 5 (71.43%)	
Failure of providing information about HIV testing		*N* = 3 (10.71%)	Yakovenko v. Ukraine (appl. no. 15825/06)
Refusal of adequate therapeutic support	Denial of antiretroviral medication	*N* = 15 (68.18%)	A.B. v. Russia (appl. no. 1439/06)
	Denial of release from detention although health status incompatible with arrest	*N* = 8 (36.35%)	
	Insufficient medical conditions	*N* = 15 (68.18%)	
	Denial of necessary care	*N* = 1 (4.55%)	
Administrative failures	Insufficient medical records	*N* = 2 (10%)	Denisov v. Russia (appl. no. 21566/13)
	Denial of investigation of medical treatment by legal system	*N* = 8 (80%)	

#### 3.4.1. Refusal of diagnostic procedures

*N* = 7 judgments (25%) dealt with cases in which applicants complained that they had been denied access to diagnostic procedures. This could mean that HIV tests were either denied altogether, that no further diagnostic consequences were drawn from positive tests (*N* = 2, 28.57%) or that examinations necessary in the context of HIV infection (such as determination of the number of CD-4 cells) were not carried out (*N* = 5, 71.43%).

An example in this category is the case Logvinenko v. Ukraine (appl. no. 13448/07). The applicant is a Ukrainian national serving a life sentence after being arrested on suspicion of murder. Already prior to his detention, he was tested positive for HIV and tuberculosis. He complained that the physical conditions during his detention and the medical assistance provided were grossly inadequate with respect to the state of his health. He was not offered any HIV-treatment and he was denied necessary blood tests to establish the number of his CD-4-cells. Moreover, he did not receive prompt, regular and adequate treatment for his tuberculosis. The physical conditions of his detention were more than dissatisfactory. Recommendations of the physicians were not obeyed by the authorities and numerous complaints of the applicant in this regard were ignored. In the view of the ECtHR, a proper treatment of the applicant’s diseases as well as regular examinations of his physical condition would have been indispensable. The court explicitly noted the denial of tests to establish the applicant’s count of CD-4 cells for numerous years in a row and judged the lack of treatment regarding the applicant’s HIV as unacceptable. In particular, it criticized the fact that tests of the level of CD-4-cells were not performed. Especially with respect to the applicant’s tuberculosis, the unawareness of his CD-4-count and hence an adequate HIV-therapy could have impeded the tuberculosis treatment. The court also criticized the conditions in which the applicant had to serve his sentence. The ECtHR found a violation of Articles 3 and 13 of the Convention.

#### 3.4.2. Failure of providing information about HIV testing

In *N* = 3 judgments (10.71%), applicants complained that they were informed of the positive result of their HIV test only after a considerable delay.

Example: The applicant in the case Yakovenko v. Ukraine (appl. no. 15825/06) had been arrested on suspicion of burglary. Among other complaints, the applicant mentioned unbearable conditions regarding his detention and the lack of medical assistance. Although his health deteriorated, the officials did not consult a physician, only an “acting paramedic.” When an HIV-test was performed on 14 February 2006, neither the applicant nor his mother were informed about the positive result. After a further deterioration of his health, he was taken to a hospital specializing in infectious diseases. There he was diagnosed with tuberculosis. During a second stay at the hospital from 20 April 2006 on, a new HIV-test was conducted. This was the first time the applicant was informed about his HIV-positive status. The delay in providing information about the applicant’s HIV-positive status by the prison authorities resulted in deferring of further diagnostic steps and adequate treatment. The ECtHR criticized not only the conditions regarding the applicant’s detention, which, in the view of the court, were a physical and psychological burden for the applicant but also the poor and deficient medical care. The court decidedly condemned that the applicant was not informed of his positive HIV-test right away, but learned about his HIV-status with considerable delay. The court also noted that the applicant did not receive any specific medical measures after first being tested positive for HIV. Furthermore, the court mentioned, that the applicant was registered as a HIV-patient at the local anti-AIDS-center only in May 2006. In the view of the court, the tuberculosis the applicant was suffering from, must be regarded as opportunistic disease. In its conclusion, the court stated a breach of the Articles 3 and 13.

#### 3.4.3. Refusal of adequate therapeutic support

We identified *N* = 22 cases (78.57%), in which PLHIV were denied appropriate medical treatment. In *N* = 15 cases (68.18%), applicants did not receive appropriate antiretroviral medication that would have been necessary for their treatment. In *N* = 8 cases (36.35%), the applicants were HIV-positive prisoners whose poor health was incompatible with continued imprisonment, and yet they were denied early release. *N* = 15 cases (48.18%) dealt with insufficient medical conditions that did not allow specific and adequate treatment of HIV-positive applicants. *N* = 1 case (4.55%) involved an applicant who was denied necessary care due to his HIV-positive status.

Example: The applicant in Case A.B. v. Russia (appl. no. 1439/06) was a detainee diagnosed as HIV-positive on his admission to remand prison in May 2004. Although his health status was deteriorating since October 2004 and although his symptoms were compatible with immunodeficiency, he did not receive adequate medical assistance. His treatment in the prison’s medical unit consisted of medication with aspirin, papaverine and analgesics. To his protests about lack of sufficient treatment, the prison’s administration responded with threats of confinement in a solitary cell. Even after his health status further deteriorated, he was denied any antiretroviral treatment, only receiving febrifuges and analgesics. He was informed that antiretroviral therapy was not possible in detention as no specific medication was available in the prison’s stock. Moreover, his application for a transfer to a specialized hospital was refused. In the view of the ECtHR, there were significant failures regarding the medical assistance the applicant received during his detention. Furthermore, the court stated that it is not the applicant’s responsibility to request specific treatment, but that he must be provided with adequate medical assistance. The court also noted that no further diagnostic measures had been carried out (such as CD-4-counts) to identify the applicant’s potential need for antiretroviral therapy. Taken all together, the court judged that the applicant did not receive the minimum scope of medical assistance regarding his HIV-infection and regarded this situation as inhuman and degrading. In its final conclusion, the court saw a violation of Articles 3 and 5 of the Convention.

#### 3.4.4. Administrative failures

In *N* = 10 of the analyzed cases (35.71%), applicants complained about administrative failures related to the medical treatment of their HIV-infection. Adequate access to healthcare includes, for example, adequate and complete records of treatment. If these are not available, gross errors in treatment can occur, and it becomes much more difficult for the patient to legally challenge potentially incorrect or inappropriate treatment in retrospect. Since medical records are a very important part of medical treatment, we also consider insufficient records as a failure to provide adequate access to healthcare. Medical records were insufficient in *N* = 2 cases (20%). In *N* = 8 cases (80%), the court refused to investigate and evaluate the medical treatment provided or the conditions the applicants had to endure which might have been inadequate with respect to their health status. Since we consider full participation in legal action regarding medical treatment that has taken place to be an important part of healthcare, this limitation of legal participation also represents a limitation of access to healthcare.

Example: The applicant in the case Sergey Denisov v. Russia (appl. no. 21566/13) was arrested on suspicion of having attempted to sell heroin. At the time of his arrest, he was already known to be HIV-positive for 11 years. He also suffered from penile cancer and chronic hepatitis C. Upon his arrest, all of his antiretroviral drugs had been taken from him. However, he received a regimen of antiretroviral drugs during his detention and his health status was regularly monitored through CD-4-cell testing. However, special treatment regarding the applicant’s hepatitis C was not recorded in his medical file. The applicant complained to the authorities that his antiretroviral drugs had been taken from him and that his condition should preclude detention. The authorities refused to assess the applicant’s complaints. Even though the ECtHR was not convinced that the medical assistance the applicant received during detention was inadequate, it found that the applicant’s complaints regarding improper medical treatment of his HIV-infection were not granted sufficient consideration. In the view of the ECtHR, the domestic courts, did not assess the effectiveness of the applicant’s healthcare, but only whether his detention could be prolonged. As the applicant was not able to legally evaluate the medical treatment he received, the court saw a breach of Article 13 of the Convention.

## 4. Discussion

We identified *N* = 28 judgments that dealt with access to healthcare for PLHIV. Regarding 67,067 judgments in the ECtHR’s database at the time of our search, this seems, at first glance, a vanishingly small number. However, one should not forget that the ECtHR can only be called upon when all national remedies have been exhausted: The analyzed cases represent only the tip of the iceberg because a large part of the cases do not even come to the attention of the ECtHR.

### 4.1. Countries against which applications were filed

The vast majority of the analyzed cases come from Russia and Ukraine (*N* = 21, 80.77%). Especially in Russia, the country with the largest HIV epidemic in Europe (26), the situation for PLHIV is disastrous. They suffer from stigmatization, marginalization, and discrimination and have no access to relevant prevention programs (26). In addition, there is a high level of dissatisfaction with the quality of specific healthcare as well as negative attitudes towards PLHIV among medical staff (7). Furthermore, there are major challenges for PLHIV in Russia to receive antiretroviral therapy due to various systemic factors such as high bureaucratic hurdles (27). This may also be the reason why there is a long delay between HIV diagnosis and the start of therapy in Russia, a general disengagement from therapy and a rapid increase in HIV-associated mortality over the last 20 years (27, 28). In Ukraine, the situation of PLHIV is also far from optimal: only 62% of adults with HIV receive antiretroviral therapy and only 75% know their HIV-status (29). In comparison, almost 75% of adults with HIV worldwide receive antiretroviral therapy and 85% know their HIV-status (30). The situation is even more favorable for Western Europe and North America, where 82.61% of PLHIV receive appropriate therapy (30). If we look at Germany alone, we even find that 96% of PLHIV receive antiretroviral medication (31). It is therefore not surprising that most of the verdicts included in our analysis come from Russia and Ukraine. In Ukraine, the situation for PLHIV has deteriorated significantly since the beginning of the Russian invasion: access to healthcare has declined even further, supplies of antiretroviral medication are dwindling and other forms of support for PLHIV have to be severely cut back (32). Regarding these two countries, there is clearly an enormous need to improve access to healthcare for PLHIV. Considering the mentioned massive discrepancies regarding the healthcare situation of PLHIV compared to other countries, it can be seen as an ethical obligation to ensure improvements. In our opinion, an ethical obligation to help and support also applies to those countries in which the healthcare of PLHIV seems to be significantly better.

### 4.2. Demographic characteristics of applicants

A striking finding at first sight regarding the PLHIV in the cases we analyzed is that *N* = 57 of the individuals (85.07%) are prisoners. This is all the more remarkable because our study did not explicitly focus on the identification of people in captivity. However, this is not surprising considering that prisoners are one of the most vulnerable groups among PLHIV. Reasons for this are a lack of knowledge (33–36), a lack of prophylaxis and often occurring examples of sexual abuse, unprotected sex or intravenous drug use. Moreover, prisoners frequently underestimate the risk of infection and the healthcare system in prisons is often inadequately equipped for their needs (37). Interestingly, the challenges for PLHIV in receiving adequate healthcare are not limited to the period of incarceration: having been incarcerated has also been shown to be a risk factor for suboptimal access to antiretroviral therapy (38). Unfortunately, prisoners face a lengthy application process if they want to claim their rights in court, and the impact of ECtHR judgments on the situation of prisoners is limited, although not entirely insignificant (39). The analyzed cases are therefore only a few examples of a successful case application for prisoners. Nevertheless, they provide an important insight into the situation of PLHIVin prisons, in particular showing the disastrous situation of prisoners in Russia and Ukraine, which seems to be in urgent need of improvement.

### 4.3. Violation of articles of the European Convention on Human Rights

In the majority of cases (*N* = 24, 92.86%), the ECtHR found a violation of at least one Article of the European Convention on Human Rights. This can be seen as a reflection of the high sensitivity the ECtHR displays towards restrictions to access to healthcare for PLHIV. Such restrictions are considered “inhumane and degrading” by the ECtHR (see A.B. v. Russia, appl. no. 1439/06) and have been clearly condemned in all the analyzed cases. The article most frequently found to be violated in our analyzed judgments is Article 3 (prohibition of torture, violation found in 71.43%). In view of the many prisoners among the PLHIV who played a role in the judgments we analyzed, this is not surprising: in its guide on Article 3, the ECtHR explicitly mentions that it is a violation of Article 3 if a person in captivity does not receive adequate medical care. The ECtHR makes the distinction that it is not sufficient for a detainee to have been seen by a doctor and prescribed a particular treatment, but that adequate healthcare includes the keeping of a medical record, prompt diagnosis and care, regular supervision, a comprehensive therapeutic strategy and conditions that permit appropriate treatment (40). In this respect, the frequent violation of Article 3 in the cases we analyzed also reflects the restrictions regarding healthcare PLHIV receive in captivity. The fact that the ECtHR’s rulings can be taken as ethical guidance in relation to various Human Rights violations underlines the high ethical obligation to help PLHIV gain adequate and unrestricted access to healthcare. However, this is not limited to the countries from which the analyzed cases originate but is to be understood as a universal ethical impetus directed at PLHIV worldwide.

### 4.4. Categories of restricted access to healthcare

Access to healthcare for PLHIV was found to be limited in different ways in our analysis. We were able to identify four main categories, with the refusal of adequate therapeutic support being by far the most significant category with *N* = 22 cases (78.57%). Here, we subsumed not only a refusal of adequate medical conditions (*N* = 15, 68.18%) or the refusal of antiretroviral medication (*N* = 15, 68.18%) but also the refusal to release a prisoner from imprisonment although his state of health did not allow him to remain in prison (*N* = 8, 36.35%). In the assessment of the ECtHR, the refusal of provision of antiretroviral medication for a PLHIV is a serious breach of Human Rights. This also applies to situations, in which a HIV-diagnosis is not communicated immediately after discovery but only after a delay so that the appropriate therapeutic and diagnostic steps cannot be initiated instantly. This situation occurred in *N* = 3 (10.71%) of the analyzed cases. Furthermore, in *N* = 7 cases (25%) we found a refusal of an HIV-test or of diagnostic consequences of a positive HIV-test, or a refusal of necessary medical examinations in the context of HIV-infection, such as the determination of the number of CD-4 cells (see Logvinenko v. Ukraine, appl. no. 13448/07). The immediate start of antiretroviral therapy in the case of newly diagnosed HIV-infection not only delays the onset of the disease and reduces the occurrence of primary and secondary consequences, but also reduces the extent of further transmission (41). Therefore, it is the clear recommendation of the WHO that antiretroviral therapy should be started immediately for all PLHIV, regardless of clinical condition and CD4-cell count, as soon as patients allow it (42). The lack of such action, as observed in the analyzed cases, not only violates the right of PLHIV to unrestricted healthcare but also contributes to a worse prognosis with regard to their disease. The ECtHR’s rulings underline the moral obligation to help all PLHIV gain adequate access to healthcare, regardless of their social status. The fact that this does not apply to people in detention is a ground for concern. In our view, it would be the task of every legislation to consistently pursue such Human Rights violations and to exert all available pressure on the respective countries in order to put the vulnerable group of PLHIV on a much more equal footing with those without HIV-infection. In *N* = 10 cases (35.71%) we found administrative failures contributed to diminished healthcare of PLHIV. These included incomplete medical records or the denial of the right to seek legal investigation into their medical care. In our view, such administrative aspects are not infrequently overlooked in research on the topic of access to healthcare. This is regrettable, as full participation in healthcare also includes upholding appropriate administrative obligations and assurance of patients’ rights in legal disputes regarding their healthcare. This applies particularly to people in detention, whose rights to freedom are severely restricted and who therefore require special support in legal proceedings.

We believe that restricted access to healthcare should be understood as a form of discrimination and stigmatization of PLHIV, regardless of whether or not Article 14 (prohibition of discrimination) was applied in the cases we analyzed in this article. This is important to consider because HIV-related discrimination is itself associated with the risk of further deterioration in the health status of PLHIV. It has been shown that HIV-related discrimination is associated with depressive symptoms and alcohol abuse (43, 44) and leads to increased psychological stress and a reduced overall health-related quality of life (14, 45). However, stigmatization experiences also have a reverse effect that in turn worsens access to healthcare for PLHIV. HIV-stigma has been shown to be a factor that encourages people not to get tested for their HIV-status. They do this in order to escape stigmatization and discrimination (46). This creates a vicious cycle, which we believe could be efficiently broken by providing unrestricted access to healthcare for PLHIV.

HIV has been a part of our reality for more than 30 years. However, the provision of adequate information on HIV-infection is still lacking and those affected still suffer significantly from stigmatization, discrimination and limited access to healthcare (47, 48).

### 4.5. Limitations

The results of this research need to be viewed in light of its limitations. First, the analyzed judgments represent only cases that were submitted to the ECtHR. A case needs to be considered in all national legal instances before an application to the ECtHR is possible. Thus, an analysis of ECtHR judgments can never show the whole picture in terms of Human Rights violations, but only the tip of the iceberg. Secondly, the categories into which we have placed the restrictions on access to healthcare for PLHIV are not exclusive but overlapping. However, in our opinion, this does not significantly limit their meaningfulness. Moreover, it seems natural that with such complex phenomena as the restriction of access to healthcare, no clearly delimited categories can be defined without omitting important aspects of reality. Finally, it might be seen as a limitation that we only focused on “HIV” in our search algorithm and did not explicitly search for “AIDS.” As explained, however, it was our concern to shed light on the special situation of PLHIV, who do not necessarily show visible external signs of their disease.

## 5. Conclusion

We looked at access to healthcare for PLHIV through an analysis of ECtHR judgments. PLHIV still experience severe restrictions on their access to healthcare, be it through refusal of diagnostic procedures, failure of providing information about HIV testing, refusal of adequate therapeutic support or administrative failures. In order to achieve the UNAIDS targets for 2025, this deplorable situation urgently needs to be improved, be it on a political or legal level. With 80.77%, most of the judgments analyzed came from the countries Russia and Ukraine. Our article shows a high need and an ethical obligation for improvement regarding access to healthcare for PLHIV. Measures should also be taken against discrimination and stigmatization of this vulnerable group. A large proportion of PLHIV in the cases analyzed were prisoners. Since limited access to healthcare for PLHIV may not be the only human rights violation faced by people in captivity, their situation should be given more attention in medical ethics research.

## Data availability statement

The original contributions presented in the study are included in the article/supplementary material, further inquiries can be directed to the corresponding author.

## Author contributions

TS-E, MO, and FS conceptualized the topic and scope of the research. TS and MO performed the data analysis, wrote, and reviewed the original draft. FS supervised the research and reviewed the manuscript. All authors contributed to the article and approved the submitted version.

## Conflict of interest

The authors declare that the research was conducted in the absence of any commercial or financial relationships that could be construed as a potential conflict of interest.

## Publisher’s note

All claims expressed in this article are solely those of the authors and do not necessarily represent those of their affiliated organizations, or those of the publisher, the editors and the reviewers. Any product that may be evaluated in this article, or claim that may be made by its manufacturer, is not guaranteed or endorsed by the publisher.

## Abbreviations

PLHIV = people living with HIV
ECtHR = European Court of Human Rights
